# Cholecystitis on gallbladder duplication: A case report and literature review

**DOI:** 10.1016/j.ijscr.2020.06.053

**Published:** 2020-06-12

**Authors:** Mabrouka Boukoucha, Fatma Dhieb, Raoudha Ben Khelifa, Hakim Znaidi, Rany Elaifi, Alifa Daghfous

**Affiliations:** a Department of Radiology, Burns and Trauma Center, Street 1er-Mai 2013, Ben Arous, Tunisia; b Department of Surgery, Burns and Trauma Center, Street 1er-Mai 2013, Ben Arous, Tunisia

**Keywords:** Gallbladder duplication, Cholecystitis, Cholecystectomy, Case report

## Abstract

**Introduction:**

Gallbladder Duplication is an uncommon anomaly of the biliary system that rarely seen during the routine ultrasound examination. It is a challenge that can be encountered by surgeons and associated with an increased risk of complications after cholecystectomy.

**Case presentation:**

We report the case of a 58-year-old woman, presented to the emergency department with persistent right upper quadrant pain. Ultrasound exam detected Gallbladder Duplication with lithiasic cholecystitis in both of them. Besides, a Computed Tomography and a Magnetic Resonance Imaging confirmed the diagnosis showing a hepatic abscess by contiguity. The Patient was operated and, three days later, she was discharged home without incident.

**Discussion:**

Gallbladder duplication is an anatomic biliary variant, which can associate with biliary ducts malformation. It can complicate the diagnosis and the surgical management of symptomatic Cholelithiasis or Cholecystitis. So, defining the exact anatomy of the biliary tree before surgery is crucial. Currently, the imaging technique, widely used for evaluating of the biliary tree, is the Magnetic Resonance Cholangiography. Laparoscopic cholecystectomy is the treatment of choice.

**Conclusions:**

Duplication of the gallbladder that could now be detected preoperatively by imaging should always be in a surgeon’s mind, since it has been associated with anatomical biliary and vessels variations.

## Highlights

• Anatomical variants of biliary tree considered as the most important predisposing for iatrogenic bile duct injuries during cholecystectomy.
• Knowledge of variant gallbladders is of an extreme surgical importance in the areas of operating abdomen procedure.
• Especially this variant can be associated with vessels variations.

## 1 Introduction

Gallbladder Duplication is an extremely rare congenital anomaly, occurring with an incidence of 1 in 4000 in the human population [1]. It is an anatomic biliary variant which is frequently discovered during surgery or at autopsy and is considered as one of the most important predisposing factors for iatrogenic bile duct injuries during cholecystectomy. So, knowledge of variant gallbladders is of an extreme surgical importance in the areas of operating abdomen procedure and it should be kept in mind by clinicians to avoid complications and prevent mistakes due to the lack of carefulness. However, anatomical evaluation of the biliary tree, that is possible now by preoperative imaging study, is crucial.

Besides, illustrating this case and reviewing the previously reported cases, the aim of this paper is to describe this rarely variant, specify the contribution of imaging in preoperative detection of this anomaly and discuss the surgical management methods. This work has been reported in line with the SCARE criteria [2].

## 2 Presentation of case

A 54-year old woman, without any past medical history, who presented to the emergency department with sharp right upper quadrant abdominal pain progressing since a week. This pain was mainly localized in upper right side abdomen and has spread towards the right shoulder. It was associated, occasionally, with nausea but no vomiting. According to the patient, she had similar episodes of abdominal pain, several months before, but recently, this pain was increasing in frequency and severity.

On admission, her vital signs were normal with blood pressure of 120/70 mmHg and pulse rate of 75 beats per minute. The physical examination showed right upper quadrant tenderness with positive Murphy sign. But no fever (37, 5°), jaundice or abdominal hernias were noted. Blood analysis found elevated white blood cells count (14,000/ μl) with negative C reactive protein (< 5). Total bilirubin level (1, 1 mg/dl), liver function (SGOT: 20UI/l, SGPT: 26UI/l) and lipase (120 UI/l) were negative.

**Fig. 1:**
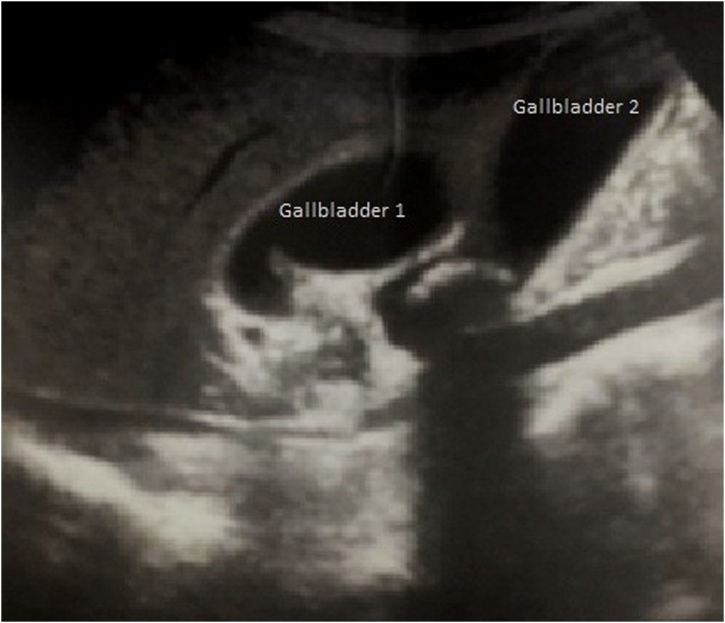
Ultrasonography findings showing two separate gallbladders (Gallbladder A and Gallbladder B) in fossa area.

The requested abdominal Ultrasonography revealed two separate gallbladders. Both gallbladders (A and B) were distended, with a thickened wall and a gallstone impacted in the infundibulum in the first gallbladder and a fundic gallstone in the second one (Fig. 1). Nearby, a multilocular collection in the hepatic segment IV was detected. On the basis of ultrasound findings and clinical symptoms, a lithiasic complicated cholecystitis on gallbladder duplication was made.

**Fig. 2:**
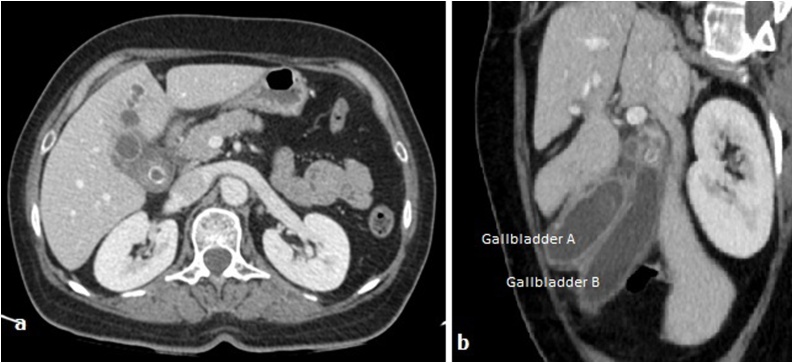
(**a**) Computed tomography scan showing lithiasic cholecystitis complicated by abscess in segment VI of the liver. (**b**) The sagittal 2D reconstruction displaying two gallbladders (A and B) with a common peritoneal coat.

**Fig. 3:**
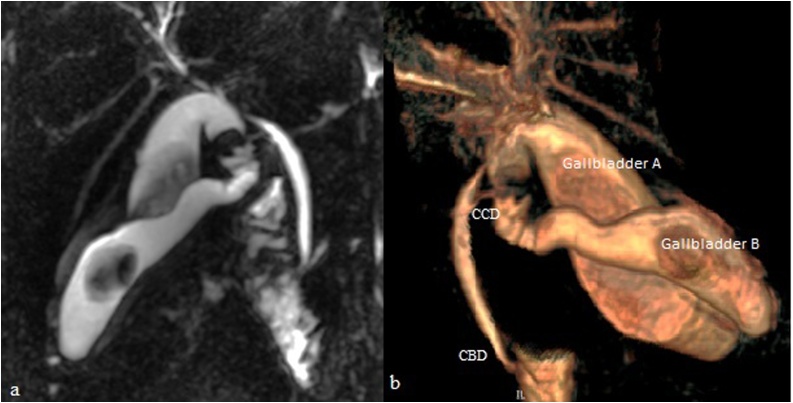
Magnetic resonance cholangiography: (**a**) Gallbladder duplication, each with one gallstone and one separately cystic duct. (**b**) Volume rendering reconstruction showing that these two gallbladders are drained by a common cystic duct (CCD) into a common bile duct (CBD).

An abdominal Computed Tomography was done, confirming the cholecystitis diagnosis and showing also a wall perforation of one of gallbladders with pericholecystic abscess (Fig. 2). For anatomical analysis of the biliary tree, a Magnetic Resonance Imaging was performed. So, findings confirmed the cholecystitis on gallbladder duplication complicated with a liver abscess. Each gallbladder had an independent cystic duct that joined in a common cystic duct before entering into the common bile duct. Furthermore, only one cystic artery was detected (Fig. 3).

Subsequently, the final diagnosis was a lithiasic cholecystitis on gallbladder duplication complicated with hepatic abscess. To reduce inflammatory sites, the patient was put on antibiotic therapy for a week. An ultrasound control before surgery showed a regression of abscess. Intervention was decided and a laparoscopic cholecystectomy was planned.

Intra-operatively, the vesicular compartment was the seat of two gallbladders enveloped in a single peritoneal membrane. The two gallbladders were lithiasic and thick-walled. In front of the impossibility of grasping the gallbladder and the difficulty in identifying the cystic duct, the surgeons decide to convert to an open operation.

**Fig. 4:**
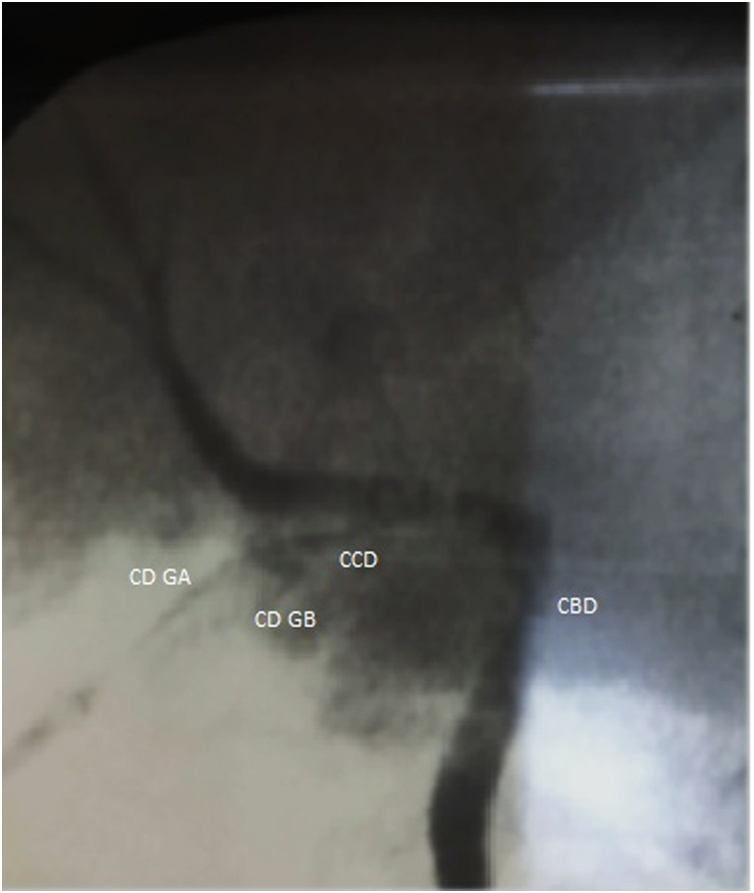
Intraoperative cholangiogram showing that each gallbladder had an independent cystic duct (CD GA: cystic duct of gallbladder A, CD GB : cystic duct of gallbladder B) that joined in a common cystic duct (CCD) before entering into the common bile duct (CBD).

**Fig. 5:**
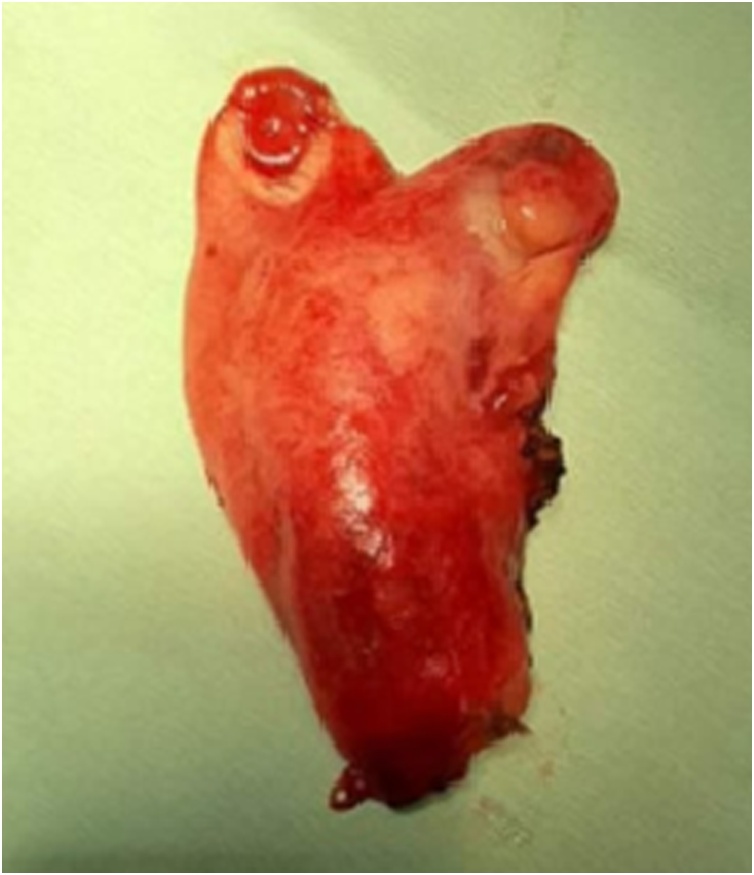
The operative piece of cholecystectomy showing two independent gallbladder in a common peritoneal coat and each with separate infundibulum.

The biliary tree dissection showed two cystic ducts that rolled one around the other with biliary leak. So an intraoperative cholangiogram was decided and an opacification of the biliary tree was done after a catheterization of the cystic duct of the first gallbladder (A). This cholangiogram confirmed the presence of a single common cystic duct opening in the common bile duct with retrograde opacification of a separate cystic duct of the second gallbladder (B) (Fig. 4). After ligature of the two separate cystic ducts a cholecystectomy with complete resection of both gallbladders was done without incident (Fig. 5). Exam of the resected specimen revealed one vesicle has a bilious content; the other was filled with a translucent liquid.

The patient had an uneventful recovery and was discharged home on post-operative day two. Within four months of follow-up, the patient is yet clinically well.

## 3 Discussion

Gallbladder Duplication is considered to be an anatomic biliary variant that its exact incidence cannot be accurately assessed. In fact, most of these anomalies have been found incidentally during surgery, imaging studies or at autopsy.

The etiopathogenisis of this biliary variant has not been clarified in a complete detail. Indeed, it is thought that is the result of excessive budding of the hepatic diverticulum during the embryonic development of the biliary tree. In 1926, and through a many cases of gallbladder duplication, collected and analyzed in man and animals, Boyden showed that there are numerous out growths and accessory vesicle formed in the 5th to 6th week of embryogenesis from a ductal system called Hepatic Antrum. Normally, these supernumerary buds regress and the persistence of one of them would result in formation of an accessory gallbladder [3,4].

Clinically, there are no specific symptoms attributable to a double gallbladder and patient can be asymptomatic or symptomatic, depending on whether it was or not complicated. The clinical significance associated with complication is similar to those encountered in a single gallbladder. Desolneux et al. reported that incidence of developing biliary diseases, such as acute or chronic cholecystis, cholangitis or adenocarcinoma is the same of only one gallbladder [5]. But some authors reported that this malformation could be associated with the development of cholelithiasis due to inadequate bile drainage [1].

For diagnosis, the most useful imaging tool is the Ultrasonography. It is able to detect gallbladder number abnormalities and to shows signs of inflammation or blockage of bile flow. But it may be limited in differentiating bilobed gallbladder from a true duplication [6]. Likewise, it is not able to define the biliary tree. Now the widely accepted imaging technique is Magnetic Resonance Cholangiography. It is superior to Ultrasonography for anatomical evaluation of the biliary tree [5,7].

Depending on configuration of cystic ducts in duplication of the gallbladder, various classifications were proposed. The most selected classification is that of Harlaftis et al., suggested in 1977, which categorized duplicated gallbladder anatomy into two main groups [8]. In type 1, having an incidence of 45.1%, the two gallbladders share a common cystic duct and the shape of gallbladders can be septated, bilobed or Y-shaped. In type 2, that has an incidence of 54.9%, called Accessory Gallbladder Group, there are more than one cystic duct joining the gallbladder with biliary tree [9].

According to Goh et al., no published literature had reported an association between a duplicated gallbladder and other duplex structures [9]. However, an association with biliary ducts, malformation or aberrant hepatic duct has been found [10,11]. Therefore, defining the exact anatomy of the biliary tree before surgery is crucial to reduce the risks of complications from biliary and vascular injury including damage to the common bile duct or other important nearby structures, in addition, to determine the type of surgical intervention laparoscopic versus open surgery.

Depending on the surgeons, laparoscopic cholecystectomy is the treatment of choice and there’s not an operating difficulties if the two gallbladders with a common peritoneal coat, or else the accessory gallbladder, is in a position other than in the normal fossa [1,12]. Congenital malformations are considered as one of the most important predisposing factors for iatrogenic bile duct injuries during cholecystectomy. Therefore, the identification of each infundibular-cystic duct junction is necessary to permit safely removing both gallbladders. A cholangiogram will differentiate both cystic ducts and make identification of the common bile duct easier [13,14].

However, some teams supports laparoscopic intervention for type 1 duplicate gallbladder and open surgery for type 2. The real reason for them is the high insertion of the second cystic duct requiring more extensive dissection, which increases the risk of injury [15].

Prophylactic surgery is not recommended for incidentally discovered asymptomatic duplex gallbladders, but the literature suggests that benefits from removing of all gallbladders are crucial once surgery is decided [16].

Literature review showed few cases of duplication gallbladder. Approximately, 20 cases were counted by Boyden until 1926, then 28 cases by Robert until 1936 [3,15]. Since, 12 cases had been reported in the literature (Table 1).

**Table 1:** Summary of all vesicular duplication reported cases published from 1926 to 2018.

Authors	Year	Cases	Type of duplication	Gallbladder stones	cholecystis	Cystic Artery / biliary variante	Operation
Boyden.E A**^2^**	Until 1926	20 cases					
Robert.E**^14^**	Until 1936	28 cases					
Udelsman**^10^**	1985	60 year old woman	Type 2	No	No	Tow cystic arteries/Anomalous right hepatic artery	Open surgery
Valdez**^6^**	2004	44 year old man	Type 1	No	No	One cystic artery	Not operated
Barut I**^13^**	2006	55 year old woman	Type 1	Yes	No	Tow cystic arteries	Open surgery
Clifford A**^5^**	2007	70 year old man	Type 1	Yes	Yes	Tow cystic arteries	Open surgery
Desolneux G**^4^**	2009	61 year old man	Type 1	Yes	Yes	One cystic artery	Laparoscopy
Causey M**^12^**	2010	15 year old girl	Type 1	Yes	Yes	One cystic artery	Laparoscopy
Shiba H**^15^**	2014	38 year old woman	Type 1	No	No	One cystic artery	Not operated
Yagan P**^1^**	2015	56 year old man	Type 1	No	no	Tow cystic arteries	Laparoscopy
Szczech E**^9^**	2015	26 year old woman	Type 1	Yes	Yes	Tow cystic arteries	Laparoscopy
Goh Y**^8^**	2015	28year old man	Type 1	No	No	One cystic artery	Laparoscopy
Ghaderi I^3^	2018	38 year old man	Type 2	Yes	Yes	Tow cystic arteries	Laparoscopy
Xavier E^11^	2018	50 year old woman	Type 1	Yes	Yes	One cystic artery	Laparoscopy

## 4 Conclusion

Duplication of the gallbladder that could now be detected preoperatively by imaging should always be in a surgeon’s mind, since it has been associated with anatomical biliary and vessels variations. Currently, the imaging technique, widely used for evaluating of the biliary tree, is the Magnetic Resonance Cholangiography. Laparoscopic Cholecystectomy with intraoperative cholangiography is the appropriate treatment in symptomatic cases.

## Declaration of Competing Interest

We have no conflicts of interest to disclose.

## Sources of funding

We have no source of funding for this reported case.

## Ethical approval

The case is exempt from ethical or ethnical approval, according to Tunisian Medical Ethics Committee.

## Consent

I, the undersigned, I have a consent for information and images concerning this medical case to be published in this article. I, the undersigned that I protected anonymity of this patient.

## Author contribution

Study conception: Mabrouka Boukoucha

Data collection: Alifa Daghfous, Fatma Dhieb, Hakim Znaidi

Writing: Mabrouka Boukoucha, Fatma Dhieb

Critical review and revision: all authors

Final approval of the article: all authors

## Registration of research studies

1. Name of the registry:

2. Unique Identifying number or registration ID:

3. Hyperlink to your specific registration (must be publicly accessible and will be checked)

This case is not interested to be registered in a publicly accessible database.

## Guarantor

MABROUKA BOUKOUCHA.

## Provenance and peer review

Not commissioned, externally peer-reviewed.
